# Knowledge, perceptions and expectations of capitation payment system in a health insurance setting: a repeated survey of clients and health providers in Kumasi, Ghana

**DOI:** 10.1186/1471-2458-13-1220

**Published:** 2013-12-21

**Authors:** Peter Agyei-Baffour, Regina Oppong, Daniel Boateng

**Affiliations:** 1 Department of Community Health, Kwame Nkrumah University of Science and Technology, Kumasi, Ghana; 2 KAMA Health Services, Kumasi, Ghana

**Keywords:** Health insurance, Capitation system of payment, Kumasi, Knowledge, Perceptions

## Abstract

**Background:**

Health insurance is improving access to quality health care in Ghana. However, there are implementation challenges which call for reform of the current health insurance system. There is no doubt that reforming the current health insurance in Ghana is besieged with a myriad of problems due to misconceptions and misinformation. This study explored the perceptions and understanding of clients and health providers on the capitation payment system in the Kumasi metropolis.

**Methods:**

The study employed a cross - sectional design and repeated surveys were conducted with a cohort of 422 NHIS policy holders aged 18–69 years in each survey. The surveys were conducted in every three months. Health service providers and clients from thirteen (13) Hospitals, seven (7) Maternity homes and twenty (20) Clinics were also interviewed. Data was collected with interviewer–administered questionnaires. STATA software (version 11) was used for cleaning, standardizing and analysing data.

**Results:**

A majority, 97.9% of the clients interviewed had heard of capitation payment although this did not translate into their level of understanding. About two-thirds, 61.2% disclosed that capitation was not important to them as clients are restricted to one Preferred Primary Provider (PPP) for a long period of time. About 94% of health providers also believed that people did not like the capitation payment system due to their misconception that it has been politicized (34%); does not give clients free choice of providers (26%) and capitation not covering most drugs (17%).

**Conclusion:**

Although awareness of the capitation was high among clients, attitudes towards the capitation payment system were somewhat poor. A good understanding of the capitation payment system is key to ensuring client and provider acceptance and smooth implementation of the system.

## Background

Health care financing in developing countries has undergone reformation over the last three decades. Since the 1980s, a large number of countries have experimented alternate health care financing mechanisms, including capitation, as methods of payment for health care providers [1]. Under the system, health care providers receive a lump sum per capita payment, intended to cover a fixed ‘basket’ of services. Capitation rates are usually based on future expenditures determined through an assessment of predictable risks or events including demographic variables, and more recently, such variables as previous diagnoses, self-reported health status and previous utilization [2]. Since no additional payment is made regardless of the services provided, the composition of the enrolled group plays an important role in determining the extent of financial risk borne by the health care providers.

In Africa and the sub-Saharan region, health service funding and use of provider payment mechanisms still remains a challenge. Ghana has experimented many health care financing mechanisms spanning from Free Health Care System (FHCS) through ‘meager’ fee for service to fee for service also called the “cash and carry” system where cost of health care was borne by patients and quite recently, health insurance. These previous systems did not survive due to many implementation challenges.

In fact, the ceaselessness of implementation obstacles to health care financing mechanisms in Ghana necessitated the introduction of the National Health Insurance Scheme (NHIS) in 2004. Since its inception, three different payment mechanisms have been introduced with the aim of improving health care as well as reducing health care cost. The scheme started with Itemized Billing (IB), where health service providers billed the NHIS per item (consumables), consultation, as well as laboratory and other services. Soon, that payment mechanism was abused and became less useful to meeting needs of the citizenry. In an attempt to address that challenge another payment mechanism known as Fee for Service (FFS) was introduced. The FFS used the Ghana Diagnostic Relation Groupings (G-DRG) where providers billed the scheme based on diagnosis with reference to a new medicines list for treatment that was introduced. Again, this system was abused. Alternative payment mechanism, capitation one of the proposed payment mechanisms in the NHI programme, became the obvious choice for experiment.

### Capitation in Ghana

The capitation system of health financing was introduced in Ghana with the support of the World Bank in 2010. The pilot scheme was done in the Ashanti Region due to its central location and heterogeneous infrastructure and culture, with one year mandate ending in 2013 after which it would be evaluated to inform roll out in the other regions of Ghana [3]. The capitation system is also expected to improve cost containment, share financial risk among scheme providers and subscribers and introduce managed competition for providers and choice for patients. Under capitation, the subscribers of the NHIS, after registration with the scheme, are asked to choose their service providers and have the flexibility to change the provider after a period of not less than six months. The capitation system is practiced alongside other provider payment mechanisms for other levels of care other than the primary level [3]. The piloting of the capitation payment system in Ghana has been met with mixed reactions from practitioners, subscribers and other stakeholders in the health industry.

Under the capitation system, the amount paid to providers caters for selected first level outpatient department (OPD) primary care cases. The expected advantages of introducing per capita payments as a complementary payment method to the already existing methods in the Ghana according to the NHIS include reducing the current high transaction cost of administrative and staff time costs of claims preparation, submission, vetting and reimbursement associated with the G-DRG and fee for services for medicines to pay for first line OPD care; improving the ability of the NHIA to forecast and budget; eliminating the current problems of delayed payment of claims – for the services in the per capita basket; reducing fragmentation of care and introducing continuity of care for clients by tying clients to a PPP of their choice; and finally enabling proper implementation of a referral system [4].

### Knowledge and utilization of health interventions

The extent of influence of knowledge and perceptions on utilization, acceptability and smooth implementation of health care interventions has been explored in previous studies [5-7]. Implementing a new system of health care financing is no exception to this. As in introduction of any new system or change, the fear of unknown and protection of interests create anxiety among parties and this may lead to misconceptions on the consequences of such new system. This study therefore aimed at assessing the expectations, perceptions and knowledge of clients and health providers on the newly introduced capitation payment system in the Ashanti region of Ghana to inform scaling up.

## Methods

### Study design

The study employed a cross-sectional study design using both quantitative and qualitative methods. This was done in order to enhance the validity of results through trangulation. The study used two different study populations. Repeated surveys were conducted with a cohort of 422 health clients bearing members aged 18–69 years per survey.

### Study setting

The study was conducted in Kumasi in the Ashanti Region, one of the 10 regions with a total population of about 4,725,046 and an annual growth rate of 3.4% as against the national growth rate of 2.8% [8]. Kumasi Metropolitan Assembly is located at almost the in the centre and often referred to as the economic nerve of Ghana. It is located in the transitional forest zone and is about 270 km north of the national capital, Accra. It is located between latitude 6.35° – 6.40° and longitude 1.30° – 1.35. The area houses all the ministries, agencies and departments of government of the region. Apart from government business it is also the hub of private businesses and enterprises. The biggest commercial centre is located in this area. It has varied health systems and a mixed of all cultures from across Ghana and abroad and currently the piloted region of NHIS capitation payment system.

### Study population and sample

All active NHIS policy holders aged 18–69 years who consented to participate in the study were eligible. The study participants or sample was drawn from across five (5) sub-metros of Kumasi. Participants were randomly selected from 40 health facilities. Non-NHIS policy holders and policy holders who did not access health care during the survey were excluded. Health service providers and clients from randomly selected thirteen (13) Hospitals, seven (7) Maternity homes and twenty (20) Clinics totaling forty (40) health facilities in the in Kumasi were involved in the study. The study used a multiple-stage stratified sampling method to select participating health facilities. Based on level of care, participating health facilities and participants were then selected from each stratum. The surveys were conducted in the months of July and October 2012. On the average, 20 participants were interviewed a day. Based on the average number of clients per day for a specific facility, and the desired sample size, a recruitment interval was developed for each facility.

Cochran’s sample size formula; $n_{0}=\frac{t^{2}*\left(p\right)\left(q\right)}{d^{2}}$ was used to estimate the sample size of 384 plus 10% non-response, 38, totaling 422 were involved in each study.

### Data collection and analysis

Two waves of survey were conducted and merged after correlation was run between the two waves which showed correlation coefficient of 0.8. All tools employed in the research were developed using standard procedures, pre-tested and revised to ensure their validity and reliability. Interviewer–administered questionnaires and in-depth interview guides were used for data collection. Information collected was socio-demographic characteristics, knowledge and prospective views of clients and health care providers on the capitation payment system. The indepth interviews were conducted with 55 health providers.

STATA version 11 was used for cleaning, standardizing data (to adjusted form), and for analysing data. The qualitative data was analysed thematically and salient quotes used to further explain the contextual meanings of the quantitative information. The reporting was based on client and provider perceptions of capitation payment system.

### Ethical consideration

The study protocols were reviewed and cleared by the institutional review board - Committee on Human Research, Publications and Ethics (CHPRE) of the Kwame Nkrumah University of Science and Technology (KNUST) and Komfo Anokye Teaching Hospital. Letters were written to the various stakeholders and opinion leaders of the study area to seek their permission and consent after explaining the intent of the study to them. The Sub Metropolitan Directors of Health Services and the Mutual Health Insurance Schemes were also contacted. Participation in the study was strictly voluntary with the informed consent of participants that guaranteed their right to privacy. Information obtained was treated with the strictest form of confidentiality.

## Results

### Background characteristics of respondents

The mean age of respondents was 36 years (SD = 14) and 34 years (SD = 12) for the first and second surveys respectively. A majority of the respondents were between 24 and 45 years. About half; 51.8% and 53.3% were married in survey one and two respectively. Thirty respondents constituting 7.2% had no formal education, 31% had secondary education whereas 29.1% had tertiary education in first survey and this was not very different from results from the second survey. The mean monthly income of respondents was GHS 482.73 (± 57.76) and GHS 413.00 (± 49.87) in first and second surveys respectively and in both surveys majority of respondents earned up to GHS 500.00 monthly. Most respondents from both surveys were employed and among respondents from the first survey, 16.4% were public servants, 23% artisans and farmers, and 29.6% were traders or businessmen. Only 7.8% were unemployed whereas 11.2% were students. Traders were however the majority of the employed in the second survey (50.9%). Majority of the health workers interviewed had tertiary education and 23.6% and 32.7% were administrators and midwives or nurses respectively. Fifty percent were junior officers, 38.9% senior staffs and 46.6% had worked with the respective facility for 2 – 5 years. Six respondents constituting 11.5% had worked with facility for more than 10 years.

### Knowledge and attitudes towards capitation payment system

Table 1 presents results of respondents’ knowledge and attitudes towards the NHIS. A majority, 89.3% of respondents knew of the benefit package of NHIS with 93% of respondents citing both laboratory and OPD services as supposed benefits. Respondents’ sources of information about their NHI package included television (18.5%), radio (47.6%), facility or providers (22%) and health workers (14.7%). Four hundred and forty-two respondents constituting 52.4% had positive impression about the NHI package whereas 18.5% had negative impression. About two-thirds, 61.1% indicated that they had both OPD and laboratory services when they visited the facility whereas 2.4% had none of these benefit packages under the NHI. More than 50% of respondents in this study were registered with the NHIS for more than two years and 53.5% for more than 3 years. A majority, 89.4% renew their policy every year and personal reasons for not renewing policy included poor service quality (44.5%), lack of money (48.2%) and preference for other sources of care (3.3%), Table 1.

**Table 1:** Summary of responses on general attitudes towards NHIS

**Variables**	**Frequency**	**Percentage**
**Know your benefit package when you visit the health facility? (n = 832)**		
– Yes	747	89.8
– No	85	10.2
**Some benefits supposed to enjoy as a NHI policy holder (n = 834)**		
– OPD services	45	5.4
– Laboratory	7	0.8
– Both	776	93.0
– None	6	0.7
Impression about NHI package*		
– Beneficial/excellent	62	7.3
– Very good	142	16.8
– Good	442	52.4
– Bad	168	18.5
– Don’t know	84	10.0
**Satisfaction with NHI package (n = 832)**		
– Yes	755	90.7
– No	79	9.3
**Which of these package(s) do you usually get? (n = 828)**		
– OPD services	296	35.8
– Laboratory	6	0.7
– Both	506	61.1
– None	20	2.4
**Which of these package(s) have you never had? (n = 815)**		
– OPD services	6	0.7
– Laboratory	95	11.7
– Both	40	4.9
– None	676	82.9
**How long have you been registered with NHIS? (n = 823)**		
– <1 year	34	4.1
– 1-2 years	151	18.3
– 2-3 years	153	18.6
– >3 years	495	60.1
**Do you renew your policy every year? (n = 826)**		
– Yes	741	89.7
– No	85	10.3
Personal reasons for not renewing*		
– Poor service quality	354	44.5
– Lack of money	384	48.2
– Taste for other sources of care	26	3.3
– Do not get sick	32	4.0
Reasons why others will not renew*		
– Poor service quality	270	32.0
– Lack of money	440	52.1
– Taste for other sources of care	36	4.3
– Do not get sick	129	14.2

(*) = multiple response.

Table 2 presents results of awareness and attitudes towards capitation payment system among respondents. Eight hundred and twenty-two clients constituting 97.9% had heard of capitation payment system and their sources of information were their Preferred Primary Provider (26.8%), radio station (54.7%) and a friend (9.2%). On exploring the understanding of clients on capitation, 7.9% believed capitation was “a payment mechanism in which providers in the payment system are paid in advance”; 10.5% also understood capitation as “A pre- determined fixed rate to provide a defined set of services for each individual enrolled” whereas 42.4% indicated its both of the above. About 28.2% however believed capitation is none of the above whereas 11% had no idea. Majority, 61.2% disclosed that capitation was not important to them as clients and their reasons included “***amount paid on behalf of clients is too small***”, “***service quality is low***” and “***capitation has a lot of problems***. The most cited reason was inability to access health care everywhere because one is restricted to one PPP.

**Table 2:** Summary of responses on awareness and understanding of captain payment system

**Variables**	**Frequency**	**Percentage**
**Heard of capitation (n = 840)**		
– Yes	822	97.9
– No	18	2.1
Source of information about capitation*		
– Preferred private provider	226	26.8
– Radio station	461	54.7
– Television station	58	6.9
– Friend	78	9.2
**Capitation is; (n = 802)**		
– A payment mechanism in which providers in the payment system are paid in advance	63	7.9
– A pre- determined fixed rate to provide a defined set of services for each individual enrolled	84	10.5
– Both	340	42.4
– Other	227	28.2
– No idea	88	11.0
**Capitation important to client? (n = 810)**		
– Yes	314	38.8
– No	496	61.2

(*) = multiple response.

On the part of health providers, they opined that capitation system of payment was very good as it would improve quality health service delivery. About 17% of the health providers indicated that their facilities had been providing services for less than 5 years under the NHIS whereas 50% said their facilities had been providing services for more than 6 years. All the health providers indicated that they renewed and would continue to renew their subscription under the NHIS every year due to the expected positive effects of the capitation system. According to health providers, clients had positive attitudes towards NHIS. However, about 77% of the providers believe some clients will not renew their policy due to low income or lack of money. A majority, 55.6% of the providers indicated that capitation was important to them because it would ensure provision of quality health care (16%), treatment continuity (19%), reducing abuse of services by clients (27%) and availability of funds prior to service provision (15%).

### Service expectations and perceived effects of the capitation payment system on service quality

**Figure 1:**
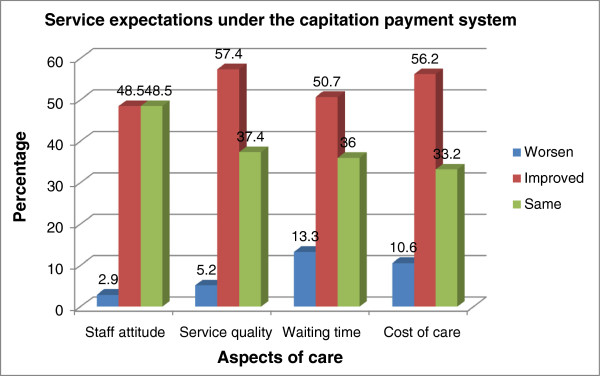
Service expectations under the capitation payment system.

Figure 1 shows results on clients’ expectations about service quality at the respective health facilities in the study area. Most respondents were expecting health service delivery to improve under the capitation system. With respect to staff attitude, 48.5% of respondents equally wanted to see an improvement or remain same. About 57% of respondents also expected an improvement in the general quality of health service whereas 5.2% expected it to worsen with the advent of the capitation system. A similar trend of expectations was observed for waiting time and cost of care. As shown in Table 3, generally, a majority of respondents had positive perception about the quality of health service under the capitation system. About 85% and 84% of respondents perceived that health staff availability and health service availability were just okay respectively. About 6.2% indicated that staff availability had worsened whereas 32.9% also believed that overcrowding at the facility was worsened and could become worst under the capitation system. About 11% and 17% of respondents also believed that the benefit package and cost of care respectively under the capitation system could worsen. However, 70.3% described prompt access to health care as just okay whereas 13.7% believed it had worsened, Table 3.

**Table 3:** Perception of quality of service under the capitation payment system

**Quality variable**	**Greatly improved**	**Improved**	**Just okay**	** Worsen**	** Worst**
Staff availability (n = 804)	4 (0.5)	54 (6.7)	686 (85.4)	50 (6.2)	8 (1)
Staff reception (802)	18 (2.2)	210 (26.2)	472 (58.9)	88 (11.0	14 (1.8)
Service availability (n = 801)	10 (1.3)	42 (5.2)	674 (84.0)	58 (7.2)	18 (2.2)
Overcrowding (n = 802)	38 (4.7)	202 (25.2)	250 (31.2)	264 (32.9)	48 (6.0)
Benefit package (n = 800)	2 (0.3)	30 (3.8)	664 (83.0)	90 (11.3)	14 (1.8)
Cost of care (n = 800)	14 (1.8)	62 (7.8)	572 (71.5)	136 (17.0	16 (2.0)
Prompt access to care (n = 802)	8 (1.0)	100 (12.5)	364 (70.3)	110 (13.7)	20 (2.4)

As shown in Table 4, 83.3% of health staff were satisfied with service they were providing to clients and 96.3% indicated that clients have been accessing services with their NHI cards since introduction of capitation payment system. The providers further asserted that, capitation was likely to simplify claims processing (22.2%), improve efficiency and effectiveness of health services through more rational resource use of resources. They said it would also provide better provider – patient relationship (38.9%) and improve primary health (PHC) delivery and thus enhance access to health care and wealth (29.6%). However, about 94% of health providers interviewed believed that some people do not like the capitation payment system and believed this was due to their misconceptions that it had been politicized (34%); people were not given primary providers of their choice (26%) and capitation not covering most of the treatment or drugs (17%).

**Table 4:** Perceived effect of capitation on health provision

**Variables**	**Frequency**	**Percentage**
**Happy with current service provision?**		
– Yes	45	83.3
– No	9	16.7
**Clients assessing service with NHIS card since capitation?**		
– Yes	52	96.3
– No	2	3.7
How will capitation help providers?*		
– Simplifying claims processing	12	22.2
– Sharing financial risk between schemes, providers, subscribers and the introduction of managed competition for providers and choice for patients to increase responsiveness of the health system	20	37.0
– Improving efficiency and effectiveness of health services through more rational resource use and also better provider – patient relationship.	21	38.9
– Improving primary health (PHC) delivery and quality of care and promoting business growth & development	16	29.6
– Other	3	5.6
**Think people like capitation?**		
– Yes	3	5.6
– No	51	94.4
**Turn up of clients increased under capitation as compared to the G-DRG?**		
– Yes	3	5.6
– No	51	94.4
**Do you think capitation is an improvement on the NHIS?**		
– Yes	30	55.6
– No	24	44.4
**Do you agree that an acceptable level of quality is being offered by all primary care facilities to all members**		
– Yes	48	94.1
– No	3	5.9
**Will providers reduce the number of request for diagnostic test for their clients?**		
– Yes	9	17.7
– No	42	82.3
**Please provide reasons**		
– Because the provider would want to make profit out of the money given?	23	42.6
– Because the provider would want to outsmart the NHIA?	9	16.7
– Because the provider would want to save the reagents for the non NHIS clients?	14	25.9
– All of the above	8	14.8
**Has capitation reduced workload in terms of claims processing**		
– Yes	42	82.3
– No	9	17.7
**Extent of reduction in workload**		
– Up to 20%	12	28.6
– 30-50%	30	71.4

(*) = multiple response.

A health provider narrated;

*“We providers think capitation will help in some sort but the choice of Ashanti region as a pilot changes the whole picture. Most clients think it’s been politicized and they believe the quality of service provision will reduce” (A Midwife).*

More than 90% of the health providers also believed that turn up of clients had increased under capitation as compared to the G-DRG payment system. They believe some private facilities had stopped seeing NHIS subscribers as a result of the capitation, pushing their clients to other public health facilities. Providers who believed clients’ turn up had decreased attributed this to client’s perception of reduced quality of service; clients preferring to visit the pharmacy instead of the hospital because they believed they would not be given all the needed drugs. A majority, 55.6% believed that capitation was an improvement of the NHIS payment system whereas 44.4% did not believe this. About 18% of respondents also believed that the capitation payment system would reduce the number of profit-induced request for diagnostic tests among providers (42.6%) and providers wanting to save reagents for non-NHIS clients (25.9%), Table 4. Most providers indicated that capitation had reduced workload by about 30-50%.

## Discussion

The perception, knowledge, expectations and attitude towards health interventions are as important as the content of the intervention. In this study structured questionnaires aided by in-depth interviews were used to explore the perceptions, knowledge, expectations and attitude of health insurance policy holders (clients) and health providers towards capitation payment system in a health insurance setting in two waves of survey each conducted three months apart.

### Knowledge and attitudes towards capitation payment system

The results showed that respondents knew of the benefit package of the NHIS and most had good impression about the scheme. The most cited source of information was the mass media highlighting the usefulness of media in providing information on healthcare to clients. As evident in this study, the attractiveness of the scheme was somewhat a determinant of people deciding to enroll or not to enroll. Lack of money and poor service quality emerged the most cited reasons why other clients would not renew their insurance policy. Failure to provide quality service that meets the expectation of clients tends to erode clients confidence in the insurance scheme. This was consistent with previous studies by Jehu-Appiah et al. [9] and Boateng and Awunyor-Vitor [10] where respondents who were previously enrolled cited high cost of premiums and lack of confidence in the schemes as the main reasons for not renewing membership.

Previous studies have tried to explain the relationship between knowledge and perceptions and utilization, acceptability and smooth implementation of health care interventions [5-7]. Implementing a new system of health care financing demands an in-depth understanding of the risks and benefits associated with the programme on the part of both providers and clients. Health care managers need to understand and accept the programme in order to provide the needed support to spearhead the implementation of the programme. This to some extent is also influenced by the perception of the health care managers about the programme. Provider payment systems could be powerful tools to promote the development of health systems and achieve health policy objectives [11] and knowledge and understanding of risks and benefits by providers and clients is essential.

About 98% of respondents in this study had heard of capitation payment system. Not all respondents however had adequate understanding of what capitation was. About 40% of respondents were able to identify what capitation was in its entirety whereas 11% had no idea of capitation payment system. Client’s level of awareness and understanding of the capitation payment system influence enrollment into the NHI under the capitation system. Evidence from other studies also show that the demand side barriers to access services such as lack of knowledge may be as important as supply factors in deterring patients from utilizing services [6,7].

Clients’ attitude towards the payment system which involves the extent to which clients attach importance to the capitation system was low in this study. Clients’ reasons behind this included low per capita rate, restriction of clients to one facility (PPP), low service provision and capitation being fraught with a lot of bottlenecks. Negative perceptions about capitation payment system among clients as reported in this study was reiterated by evidence from interview with the providers who also believed that some clients do not like the capitation payment system and believed this was due to their misconception that it has been politicized, people not given primary providers of their choice and capitation having limited benefit package. These assertions are however not in line with what was stipulated by the NHIA which indicate that subscribers of the NHIS, after registration with the scheme, would be made to choose their service provider and have the flexibility to change the provider after a specific period [3].

Some 40% of the health providers also indicated that capitation was not important to them. However a majority of health providers who deemed capitation as an important health financing strategy to the existing G-DRG alluded this to the provision of quality healthcare, ensuring treatment continuity, reducing abuse of services by clients and provision of funds before service provision. Under this payment system, incentives are created for managers to control expenses while achieving their production targets. This is consistent with Hellinger [12] who indicated that Capitation discourages resource consumption. According to Hellinger [12], capitation as a population based form of payment, offers the potential for stimulating attention to epidemiological patterns of illness and care, of being buttressed by clinical protocols defining which form of care is expected in which context, and of encouraging resource-conserving practice innovations.

Goals that support the adoption of capitation payment system include creating incentives for providers to improve efficiency through more rational resource use, including increasing health promotion and disease prevention services, and supplying higher-quality services with the resources available [11]. Restricting clients to a single primary provider ensure reduction in the abuse of services and equity in health utilization yet any mistake arising from incompetency of health providers could have a continuous debilitating influence on the client as long as the client remains under that provider.

Some health providers in this study also held the views that capitation simplifies claim processing and it is improving primary health (PHC) delivery and quality of care and promoting business growth and development. Majority of health providers indicated that capitation has reduced workload to about 30-50%. Consistently, Bazzoli et al. [13] indicated that provider capitation promotes cooperation and health delivery through enhancing integration between hospitals and physicians in relation to administrative/practice management, physician financial risk sharing, joint ventures to create new services, computer linkages, and an overall measure of physician-hospital integration. This is however not congruent with [14] who believed that capitation performs poorly in terms of physician productivity and patient service. This he alluded to the fact that its payment is determined prospectively without regard to the number of services provided; overpaying physicians who stint on care and underpaying those who provided many complex services. This might expose the providers of services to extra costs and hence lower net income, for treating patients with more severe underlying disease and greater need for time and services [15].

The NHIA is inclined to the school of thought that that says that the capitation system improves cost containment, share financial risk among scheme, providers and subscribers, and introduce managed competition for providers and choice for patients [3]. Creating or strengthening PHC institutions to operate autonomously and provide comprehensive, integrated, first-contact care for individuals and the wider community is however a primary goal of the capitation payment system and this must be ensured [11]. Our study has revealed two dissenting views held by clients and health providers. Whereas a majority of clients realigned themselves with the negative view point of the capitation system, most health providers viewed the capitation positively in terms of its role in garnering resources for service provision and improving quality health care.

The study was associated with some limitations. The study was conducted in health facilities thus only clients who accessed care during the study period were interviewed. This means that majority of clients were excluded by default and valuable perception could not be solicited. The surveys were conducted three months apart. While this could capture diverse behavioural practices, the periods may have their own contextual factors that may affect respondents’ perceptions, knowledge, expectations and attitudes towards the new payment mechanism. However, with internal validity and reliability measures such as random selection of respondents, pretesting of tools and triangulation, the findings could be useful in forming roll out of the intervention in Ghana.

## Conclusion

This study shows that although awareness of capitation was very high it did not translate to adequate understanding of capitation among clients. There are two opposing schools of thought represented by clients and health staff. Not all clients and health staff deemed capitation as important. While most clients perceived the capitation payment system as bad, a high proportion of health staff consider it as very good. Both clients and health staff have high expectations from the capitation payment system. An upscale in the current and existing educational interventions to educate clients and update health staff on capitation payment scheme could enhance understanding and acceptability and would be useful in scaling up the capitation payment system nationwide in Ghana.

## Competing interests

The authors declare that they have no competing interests.

## Authors’ contribution

The study was conceived and designed by PAB and RO. RO led the data collection under supervision by PAB and data was analyzed and reported by DB. The initial manuscript was written out by DB. All authors reviewed and critically revised the manuscript for important intellectual content and agreed to submit the manuscript for publication. All authors read and approved the final manuscript.

## Pre-publication history

The pre-publication history for this paper can be accessed here:

http://www.biomedcentral.com/1471-2458/13/1220/prepub
